# Tracheocutaneous Fistula After Tracheostomy: Spotlight on a Closure Technique With a High Success Rate

**DOI:** 10.7759/cureus.39462

**Published:** 2023-05-25

**Authors:** Mariam J Aljehani, Alzargaa Tamadhor, Arwa Alkhunaizi, Jumana K Alahmadi, Ahmad Alkurdi

**Affiliations:** 1 Otolaryngology - Head and Neck Surgery, Ohud Hospital, Madinah, SAU; 2 Otolaryngology - Head and Neck Surgery, Prince Mohammed Bin Abdulaziz Hospital, Riyadh, SAU; 3 College of Medicine, Al Rayan Medical College, Madinah, SAU; 4 Otolaryngology - Head and Neck Surgery, National Guard Health Affairs, Madinah, SAU; 5 Otolaryngology - Head and Neck Surgery, Prince Mohammed Bin Abdulaziz Hospital, Madinah, SAU

**Keywords:** fistula repair, surgery, fistula, trachea, tracheocutaneous fistula

## Abstract

Tracheostomy is a commonly performed procedure in patients requiring prolonged mechanical ventilation. While it effectively provides a secure airway, tracheostomy can lead to complications, such as tracheal stenosis, tracheomalacia granulation tissue, pneumonia, aspiration tracheovascular fistula, tracheoesophageal fistula, and tracheocutaneous fistula.

In this systematic review, we aim to identify the most suitable closure method and compare recurrence outcomes between methods for persistent tracheocutaneous fistula (TCF) in all age groups. We conducted a bibliographic search between January 1st, 2011 and March 10th, 2021 in the PubMed, B-on, Scopus, and Web of Science databases, and also performed a manual search to identify relevant articles. Our inclusion criteria were case series or comparative studies of surgery for persistent TCF and the success rate of TCF closure in patients both below and above 18 years of age.

After applying the inclusion criteria to the research results, we included nine studies in our analysis. We found that closure by secondary intention is the most effective method for TCF closure, followed by primary and secondary intention combined. The success rate with most of the techniques was high, regardless of the size of the fistula or associated comorbidities, and its simplicity makes it an appealing treatment option in the care of patients with TCF.

In conclusion, this systematic review highlights the importance of selecting an appropriate closure method for persistent TCF and provides valuable insights into the success rate of various techniques.

## Introduction and background

Tracheocutaneous fistula (TCF) is a common complication that can occur in patients who have undergone tracheostomy. Although the tracheostomy site usually heals after cannula removal, a small percentage of patients, ranging from 1%-3%, may develop a chronic TCF [1]. The incidence of a fistula increases with the length of cannula use, and its occurrence has been growing due to the increasing use of tracheotomies for chronic conditions such as protracted respiratory failure and congenital deformity, instead of for acute respiratory tract infections [2]. The persistence of TCF can lead to several concerns for both the patient and their family, including hygiene, aspiration, and aesthetic issues [3,4]. Therefore, it is crucial to close or modify the TCF to avoid these complications.

The surgical treatment of TCF has been a subject of scholarly controversy [5]. Primary closure involves the excision of the fistulous tract, followed by multi-layered closure, while secondary closure permits healing by secondary intention after tract removal [6,7]. Closure of small TCFs is usually simple, safe, and performed by conducting restricted local treatments. However, the treatment of large TCFs is often complex and may result in various unpredictableconsequences [8]. Nonetheless, surgical closure is generally considered a safe and effective treatment option for many conditions, although it carries some degree of risk, such as infection, bleeding, nerve damage, adverse reactions to anesthesia, delayed healing, scarring, and recurrence of the underlying condition.

This systematic review aims to evaluate the effectiveness of various surgical closure approaches for TCF repair and the occurrence of adverse events. By conducting a comprehensive analysis of the most recent research studies, we aim to provide insight into the most effective surgical treatments for TCFs. This will help clinicians make informed decisions and select the most appropriate treatment options for their patients. By minimizing the risks of complications associated with TCF, patients can receive better care, and their quality of life can be enhanced.

## Review

Methods

Protocol and Registration

This systematic review was registered in the PROSPERO database and reported using the PRISMA-NMA (Preferred Reporting Items for Systematic Reviews and Meta-Analyses - Network Meta-Analysis) statement and the PICO (Population, Intervention, Control, and Outcomes) framework, based on the study's scope, objectives, hypothesis, and methodology.

Eligibility Criteria

The inclusion criteria for this study were - patients across all age groups who received surgical treatment for TCF. The exclusion criteria were - patient series that were duplicates, and case reports or case series that included fewer than two patients.

Information Sources and Search

To obtain relevant studies and answer the question posed by this review, the following combinations of words were used for searching in PubMed, B-on, Scopus, and Web of Science - ‘Tracheocutaneous fistula’ and ‘Tracheocutaneous closure’ - by two independent reviewers. From the database searches, articles published between 1 January 2011 and 1 December 2022 were considered for analysis. Furthermore, we conducted a manual screening of the reference lists of all the articles included in the final data set to ensure that no relevant studies were overlooked during the electronic search.

Study Selection

Two independent reviewers screened the records by title and abstract using strict inclusion and exclusion criteria to identify studies. The eligible articles were then assessed in full text by the same two independent reviewers. Only studies and series that matched the inclusion criteria were considered for inclusion in this review.

Data Collection Process

Data extraction was performed independently by two reviewers. Data were extracted regarding study information (author and year), study design, treatment, intervention, and follow-up.

Results

**Figure 1:**
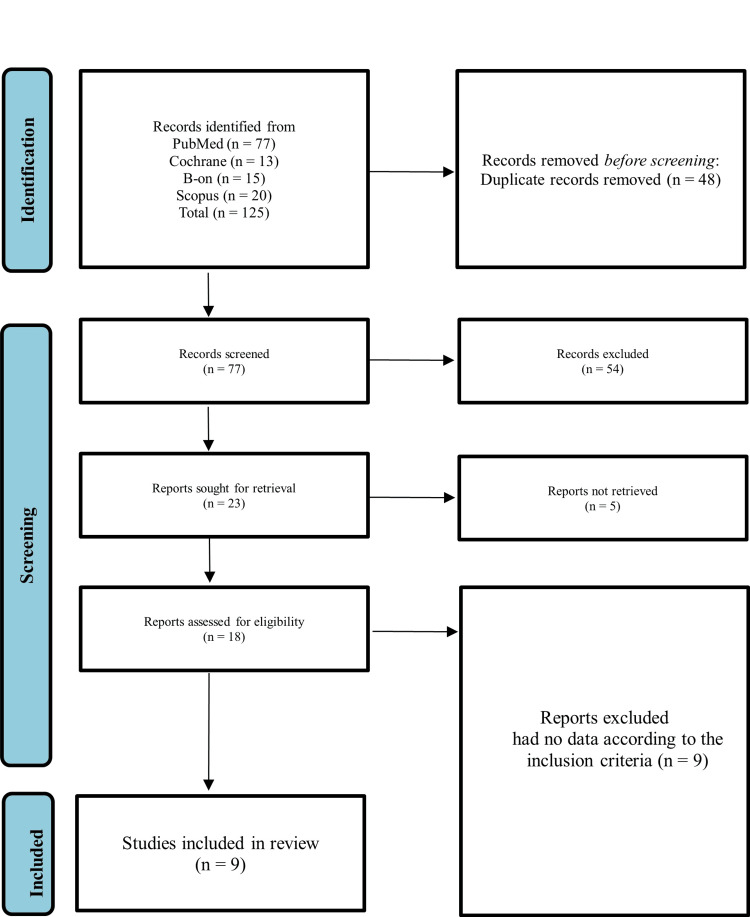
Identification of studies via databases and registers

After conducting the initial search, 125 articles were found (Figure 1). The Mendeley® citation manager (Mendeley Ltd., London, UK) was used to remove duplicates, leaving 77 articles for further analysis. These articles were screened based on their title and abstract, and 23 were deemed eligible for full-text analysis. Five full texts were unavailable and thus excluded. After the complete text analysis of the remaining 18 articles, nine were excluded, and only nine were included in the final analysis. The PRISMA flowchart depicting this process is shown in Figure 1.

Four articles included a combination of either primary or secondary closure alone, and five included secondary closure alone (Table 1).

**Table 1:** List of included studies and their details TCF: tracheocutaneous fistula

SN	Type of Study	Year of publication	Author	N	Causes of TCF	Treatment options
1	Case report	2020	Li et al. [9]	1	After thyroid tumour excision and tracheostomy	Rigid and flexible bronchoscopy combined with flap transplantation
2	Case series	2020	Vardhan H et al. [10]	3	Post thyroid surgery trauma	Complex reconstruction + primary closure for the primary site
3	Cross-sectional	2018	K.R. Feehs et al. [11].	31	Long-term intubation	Simple primary closure + turn over flap
4	Cross-sectional study	2018	Hauff et al. [12].	21	Prolonged ventilation followed by upper airway obstruction (neoplasia and obstructive sleep apnoea)	The suture-ligature technique
5	Case report	2015	Royer et al. [8].	1	Papillary thyroid carcinoma treated with total thyroidectomy	Three-stage approach + radial forearm free flap
6	Cross-sectional study	2019	Smith et al. [13]	108	Upper airway obstruction, subglottic stenosis, glottic obstruction, neurologic disease	Primary multi-layered closure over a passive rubber band drain.
7	Systematic review	2016	Lewis et al. [14]	413		Primary closure sucess rate 95.7%, secondary intention sucess rate 92.7%
8	Case report	2019	Sweeney et al. [15]	1	Severe Guillain-Barré syndrome requiring prolonged ventilation via tracheostomy	Use of silver nitrate
9	Case report	2011	M. Kamiyoshihara et al. [16]	1 case	Tracheostomy was performed during treatment for drug-induced anaphylactic shock.	Hinged skin flap

There were nine studies included in the analysis, comprising four case reports, one case series, one systematic review, and three cross-sectional studies. However, none of the cross-sectional studies had any control or comparison groups. It is noteworthy that there were no reported mortalities. The success rates for closure varied between 80% and 100% across the studies. Upon analyzing the four studies that reported both primary and secondary closure outcomes, no significant association was found between the type of closure and successful closure rates (RR: 0.99 [0.96 to 1.06]). The level of heterogeneity between the studies was minimal (t2 = 0), and there was no significant variance between the studies (Q = 1.44, P < 0.624). The heterogeneity percentage was 0% (I2 = 0%). Please refer to Table 2 for more details.

**Table 2:** Association of recurrence and closure method with the size of the defect TCF: tracheocutaneous fistula

SN	N	Size of defect	Risk factors	Operative technique	Rate of recurrence	Follow up month
1	1	1 cm below glottis (3.0 cm × 1.0 cm)	Unhealthy margins	Tracheal stent for tracheal wall + peripheral and turnover the flap + closure stoma with optimal tension + cervical defect with deltopectoral flap	0%	12 months
2	3	Different sizes	With the loss of tissue	Pectoralis major myocutaneous pedicled flap and skin flap + sternocleidomastoid musculocutaneous flap	0%	4 months
3	31	Different sizes		Simple primary closure with superiorly based turnover skin flap	0%	3–4 months
4	21	The mean fistula size was 78.3 mm^2^ (range, 8.0–314.2 mm^2^	Smokers, diabetes, history of radiation, immunocompromised	The simple technique does not require major tissue rearrangement or grafting. The suture-ligature technique of TCF closure + rubber band drain	0%	6 months
5	1	1.4 cm × 1.6 cm	Post-radiation failure of previous primary and rotational graft closure	Inner mucosal lining (buccal mucosa), a central cartilage structure (conchal cartilage), and an external skin lining were constructed on the patient’s distal volar forearm and subsequently harvested in a staged fashion. This graft was transferred as a free flap.	0%	at 6, 12, 18, and 60 months postoperatively
6	108	Different sizes	Paediatric trachea	Primary multilayered closure over a passive rubber band drain. This surgical technique affords immediate resolution of the TCF.	20%	5 months
7	413	Different sizes	Paediatric trachea	Primary closure, secondary intention	5.2%	Over 4 years
8	1	Small size	Severe Guillain-Barré syndrome	Non-surgical approach and use of silver nitrate ~4 mm into fistula & roll to cover skin edge. Repeat twice	0%	3 months
9	1	-	poor wound healing + bile duct cancer and primary sclerosing cholangitis	Closed with an autogenous hinged skin flap. The flap was sutured to the tracheal defect with 3-0 absorbable monofilament and interrupted sutures. The soft-tissue defect was covered by the anterior cervical muscles.	0%	22 months

Discussion

Based on Vardhan H et al. [10], primary or secondary closure, complex flap closure, and non-surgical techniques are perceived to have fewer adverse outcomes and complications than primary closure alone. Musculocutaneous flaps are advantageous because the muscle layer between the skin paddles provides a waterproofing layer that reduces the risk of post-operative fistula.

Lewis et al. [14] conducted a comprehensive review and meta-analysis of 14 papers involving 646 patients, comparing excision with primary closure versus excision with secondary closure. The study found no statistically significant differences between primary and secondary closure in terms of successful closure rates, rate of revision surgery, the incidence of subcutaneous emphysema or pneumothorax, urgent airway issues, wound infection, or wound dehiscence. However, cosmesis or scarring was not included as an outcome measure. This study has several significant limitations, including the absence of randomization, potential allocation bias in cohort studies, and lack of information regarding the criteria used to allocate patients to primary or secondary closure in some studies.

Feehs et al. [11] conducted a retrospective cohort analysis that showed the use of an autogenous hinged skin flap harvested from the inferior peristomal tissue and reflected superiorly to fill the tracheal defect. Kamiyoshihara listed many benefits of the turnover-hinged skin flap approach, such as less suturing and strain on the anterior cervical muscle, and fewer difficulties with flap anastomotic insufficiency [15, 16].

The turnover-hinged skin flap approach should be known and repeatable to all surgeons, and it is applicable to necks of any thickness. This procedure has a high success rate, independent of the size of the fistula or the presence of concomitant comorbidities. It is a simple and attractive therapeutic choice for patients with TCF. However, on postoperative day one, two patients (9.5%) developed subcutaneous emphysema and required reopening of the tract. These two patients were both smokers, had minor fistulae (12 and 20 mm2), and had a rubber band drain implanted during surgery [17].

Permanent/fenestrated tracheotomy involves stitching an inferiorly-based tracheal flap to the skin or subcutaneous tissues to formalize the tracheostomy [18]. The treatment begins with diagnostic laryngoscopy and bronchoscopy to determine whether the airway is adequate for TCF repair [19]. Some surgeons utilize excision of the fistulous tract with secondary intention healing to reduce the risk of postoperative subcutaneous emphysema, pneumomediastinum, and pneumothorax [20].

Persistent TCF may lead to the aspiration of liquids via the fistula and the development of pneumonia. Therefore, it is essential to close the fistula to avoid aesthetic and saliva and mucus discharge problems. The most common causes of pediatric TCFs are epithelialization, inflammation or infection, and blockage. Sweeney et al. [15] provide an additional non-surgical use of topical silver nitrate for sealing a tracheocutaneous fistula. However, topical silver nitrate administration can be complex, and it is considered a significant and less invasive therapy option for chronic tracheocutaneous fistula.

## Conclusions

In conclusion, the included studies demonstrated success rates ranging from 80% to 100% for closure procedures. The analysis did not identify a significant association between the type of closure (primary or secondary) and successful closure rates, indicating that both techniques may be equally effective in achieving closure. Additionally, the minimal level of heterogeneity among the studies suggests consistent findings across the literature. Nonetheless, further research is warranted to expand the evidence base and investigate other factors that may influence closure outcomes in different clinical scenarios.
